# Primary Extraosseous Ewing Sarcoma of the Vulva: Unravelling the Mystery of a Common Tumor in an Uncommon Site

**DOI:** 10.7759/cureus.88632

**Published:** 2025-07-23

**Authors:** Syamsritha Gondi, Venkata Renuka Inuganti, Chaitra B, Sudhakar Ramamoorthy, Santhi Imandi

**Affiliations:** 1 Pathology, NRI Medical College, Guntur, IND; 2 Pathology, Guntur Medical College, Guntur, IND

**Keywords:** cd99, ewing sarcoma (es), ewsr1 fish, extraosseous ewing sarcoma, nkx2.2 protein, small round cell sarcoma, vulvar cancer

## Abstract

Primary vulvar Ewing sarcoma (ES) is exceedingly rare, particularly in postmenopausal women. To date, only 14 cases have been confirmed through molecular cytogenetic analysis.

We report the case of a 55-year-old woman presenting with a painful, ulcerated vulvar mass. Imaging revealed a 6×7.9×8.5 cm lobulated soft tissue lesion. Contrast-enhanced computed tomography (CECT) demonstrated pulmonary metastases. Histopathology of the vulvar mass showed sheets of malignant small round cells, which were highlighted by strong membranous CD99 expression and NKX2.2 nuclear expression on immunohistochemistry. Fluorescence in situ hybridization (FISH) using a break-apart probe confirmed EWSR1 gene rearrangement, establishing the diagnosis of ES. Despite receiving eight cycles of chemotherapy, the patient succumbed to the disease.

This is the 15th reported case of primary vulvar ES with molecular confirmation. The case highlights the importance of considering ES in the differential diagnosis of vulvar tumors and underscores the value of molecular diagnostics in guiding management.

## Introduction

Ewing sarcoma (ES) is a malignant small round blue cell tumor belonging to the Ewing sarcoma family of tumors (ESFT). It is characterized in most cases by the chromosomal translocation t(11;22)(q24;q12), which results in EWSR1::FLI1 fusion transcript in 85-90% of cases. The second common molecular alteration noted is t(21;22)(q22;q12), which results in the EWSR1::ERG fusion [1].

Although primarily a bone tumor affecting adolescents and young adults, approximately 20-30% of ES cases present as extraosseous soft tissue tumors [2]. Among them, ES of the female genital tract is exceedingly rare, with isolated case reports involving the cervix, vagina, and vulva being published. To date, only 14 cases of primary vulvar ES have been confirmed by molecular studies [3-5].

ES in older adults is extremely uncommon, with studies reporting that only approximately 11.8% of all ES cases occur in individuals over 40 years of age and prognosis in that age group is significantly poorer [6]. We present a rare case of primary vulvar ES in a 55-year-old woman, confirmed by immunohistochemistry (IHC) and molecular cytogenetics. We also discuss its clinical implications and review of literature.

## Case presentation

A 55-year-old postmenopausal woman presented to the emergency department with acute pain and an ulcerated mass over the left labia. Clinical examination revealed an irregular, lobulated, immobile mass measuring approximately 9×8×3 cm, with a 5×4 cm central ulcer showing raised edges. She had no significant past medical or surgical history.

**Figure 1:**
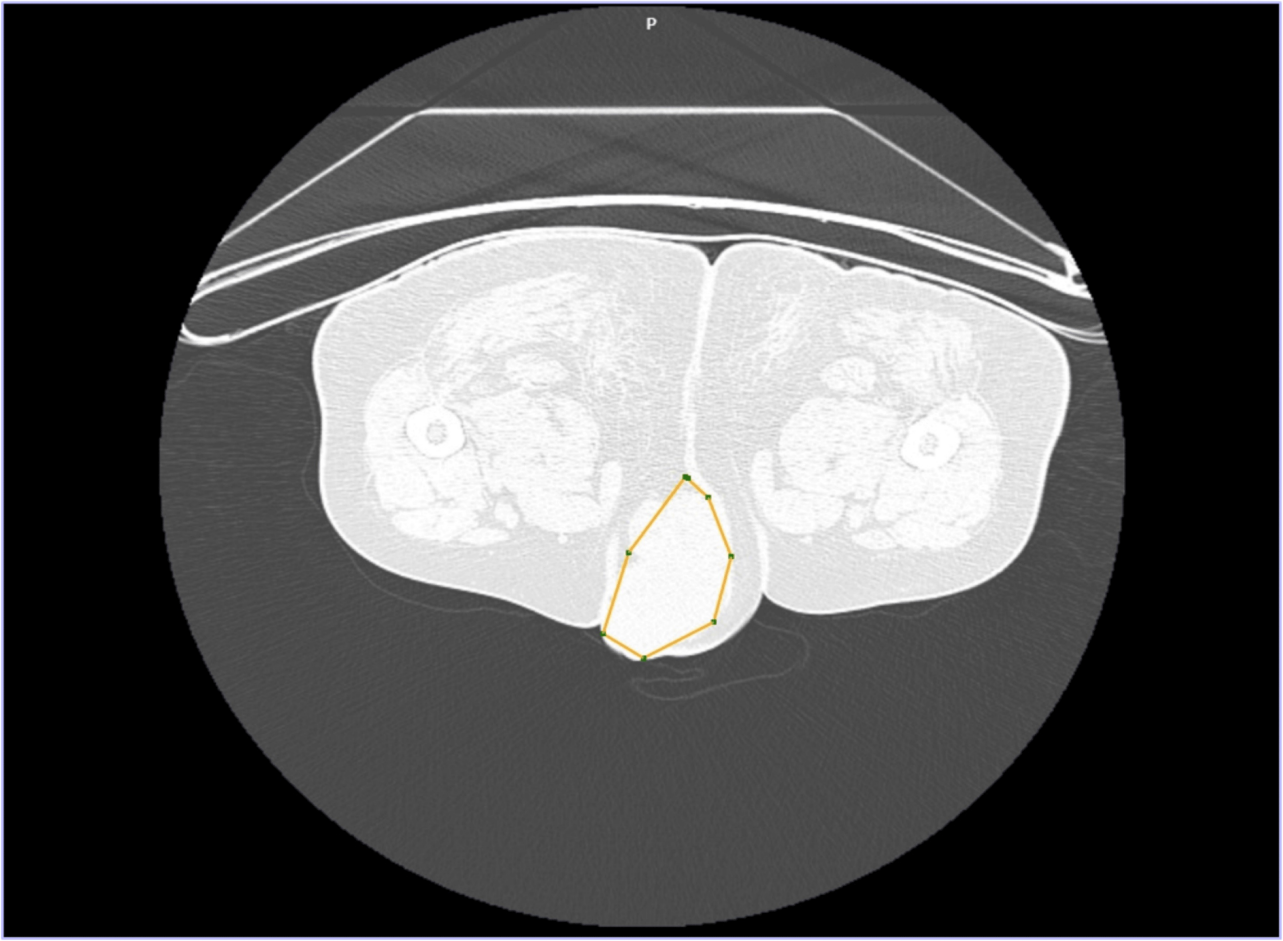
CECT showing a large ill-defined lobulated soft tissue density lesion noted arising from the vulva measuring 6×7.9×8.5 cm CECT: contrast-enhanced computed tomography

Initial blood investigations revealed anemia (hemoglobin 7.3 g/dL) and marked leukocytosis (total leukocyte count 48,560/cumm). Contrast-enhanced computed tomography (CECT) showed a 6×7.9×8.5 cm ill-defined lobulated soft tissue lesion arising from the vulva (Figure 1).

**Figure 2:**
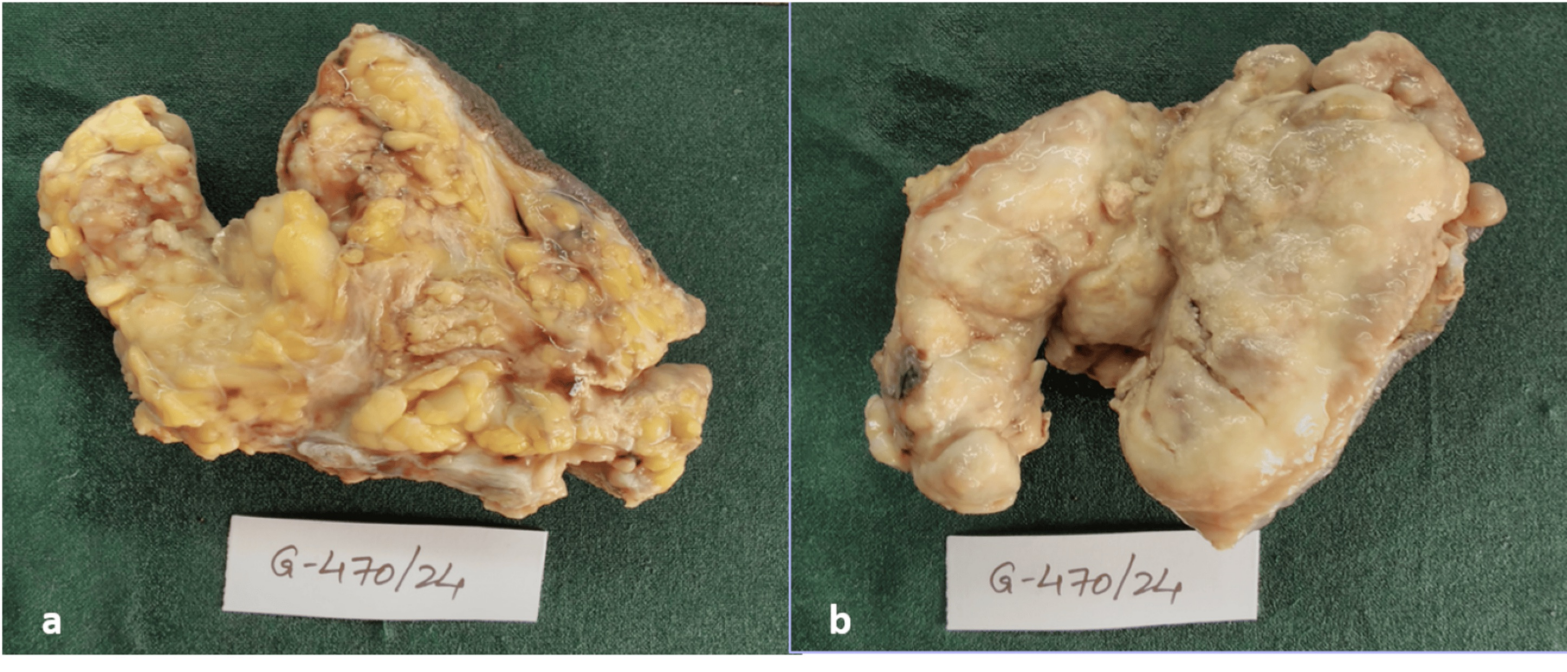
(a) Gross photography showing the external surface of the vulvar mass. The tumor is skin covered. (b) Cut section of the tumor revealing a nodular, grey-white, and firm cut surface

The mass was excised with wide local resection and submitted for histopathological evaluation. On gross examination, the specimen measured 9×8×7 cm and was partly skin covered with ulceration. Cut section revealed a nodular grey-white and firm mass (Figure 2).

**Figure 3:**
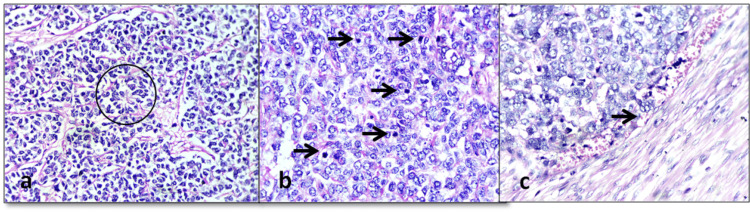
Light microscopy: (a) Tumor cells arranged in sheets and islands separated by fibrovascular septae. Few rosettes are also noted (H&E ×100). (b) Tumor cells exhibit frequent typical and atypical mitotic figures (H&E ×200). (c) Lymphovascular invasion noted at the tumor invasive front (H&E ×200) H&E: hematoxylin and eosin

Microscopically, the tumor was located in the subcutaneous plane, composed of uniform small round cells arranged in sheets and islands, with occasional rosette formation (Figure 3a). The tumor cells exhibited a high nuclear-to-cytoplasmic ratio, finely stippled chromatin, inconspicuous nucleoli, scant cytoplasm, and frequent mitotic figures (>20/10 HPF) (Figure 3b). Lymphovascular invasion was also observed (Figure 3c).

**Figure 4:**
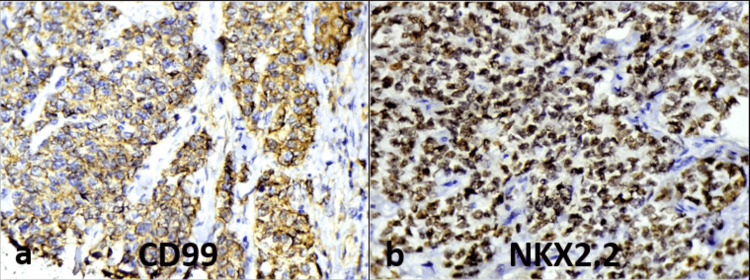
Immunohistochemistry: (a) CD99 IHC staining, diffuse strong membranous positivity in tumor cells (×400). (b) NKX2.2 IHC staining, diffuse strong nuclear positivity in tumor cells (×400) IHC: immunohistochemistry

IHC demonstrated strong diffuse membranous positivity for CD99 (Figure 4a) and nuclear positivity for NKX2.2 (Figure 4b). The tumor cells were negative for pancytokeratin, chromogranin A, and HMB45, effectively excluding carcinoma, neuroendocrine tumor, and malignant melanoma (Table 1). These findings strongly supported a diagnosis of ES.

**Table 1:** Summary of IHC markers IHC: immunohistochemistry

IHC markers	Result	Diagnosis
Pancytokeratin	Negative	Ruled out: Poorly differentiated carcinoma
Pancytokeratin, chromogranin A	Negative	Ruled out: Neuroendocrine carcinoma
HMB45	Negative	Ruled out: Amelanotic melanoma
CD99, NKX2.2	Positive	Final diagnosis: Ewing sarcoma

Further, molecular testing was performed using break-apart fluorescence in situ hybridization (FISH) for the EWSR1 gene. The test revealed rearrangement in 95% of tumor cells, confirming the diagnosis of ES.

**Figure 5:**
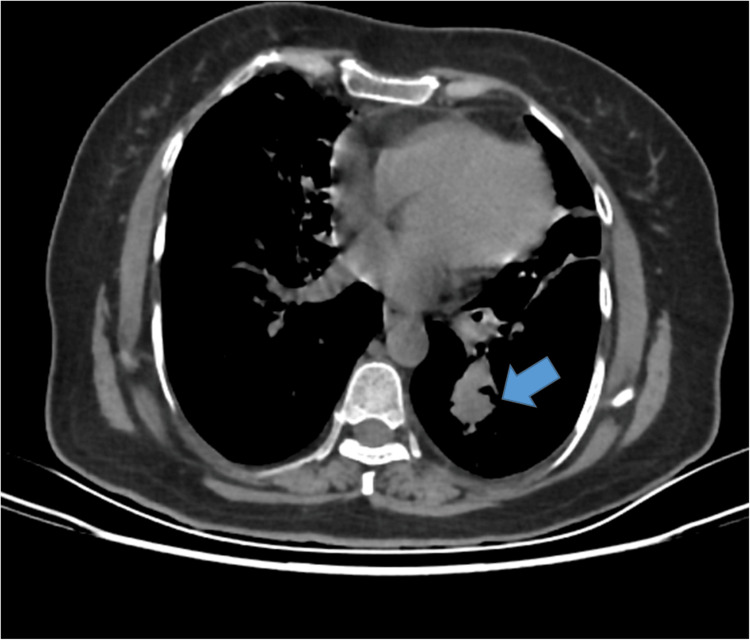
CECT of the thorax showing ill-defined soft tissue density in the left lung suggestive of pulmonary metastasis CECT: contrast-enhanced computed tomography

A subsequent CECT identified ill-defined soft tissue density lesions in the superior and posterior basal segments of the left lower lobe of the lung, with nodular septal thickening suggestive of pulmonary metastasis (Figure 5).

The patient was initiated on VDC/IE (vincristine, doxorubicin, cyclophosphamide/ifosfamide, etoposide) chemotherapy regimen and received eight cycles. Unfortunately, despite treatment, her clinical condition deteriorated, and she succumbed to the disease nine months after diagnosis. 

## Discussion

Demographics and site

ES predominantly affects adolescents and young adults, with the highest incidence occurring between 10 and 20 years of age. Its occurrence in older adults, particularly those over 50 years, is exceptionally uncommon [6,7]. In a study by Karski et al., the incidence of osseous ES in patients aged 40-60 years was reported to be 37%, which was significantly lower compared to 67% in patients younger than 20 years of age [8].

Extraosseous ES is even rarer, accounting for approximately 12% of all ES cases [9]. These tumors most commonly arise in the trunk, lower extremities, and paravertebral regions, exhibiting aggressive behavior with a high propensity for metastasis to the lungs, bone, and bone marrow [1,10]. Involvement of the female genital tract is exceedingly rare, with case reports in the ovaries, cervix, vagina, and vulva. Among these, vulvar ES is the least frequent, with only 14 cases confirmed by molecular analysis documented in the literature to date [4,5,11].

Diagnostic challenges

Histologically, ES must be differentiated from other small round blue cell tumors, including amelanotic melanoma, neuroendocrine carcinoma, rhabdomyosarcoma, lymphoma, and desmoplastic small round cell tumor. IHC plays a crucial role in narrowing down the differential diagnosis (Table 2) [12].

**Table 2:** Differential diagnosis of small round blue cell tumors in the vulva

Differential diagnosis	Histology	Immunohistochemistry
Amelanotic melanoma	Atypical melanocytes with prominent nucleoli	HMB45+, melan A, S100+
Neuroendocrine carcinoma	Small uniform round cells with frequent mitosis and karyorrhectic debris	Chromogranin A+, synaptophysin+, CD56+
Rhabdomyosarcoma	Strap cells, rhabdoid morphology	Desmin+, myogenin+, MyoD1+
Lymphoma	Monotonous round cells, high mitoses	CD45+, CD20+ (B cell), or CD3+ (T cell)
Desmoplastic small round cell tumor	Small round cells with desmoplastic stroma	Cytokeratin+, desmin+, WT1+, EWSR1 gene rearrangement
Ewing sarcoma	Sheets/rosettes of small round cells	CD99+ (diffuse membranous), NKX2.2+, EWSR1 gene rearrangement

CD99 is highly sensitive but not specific. Though positive in other tumors, diffuse membranous expression of CD99 supports the diagnosis of ES. NKX2.2, a transcription factor, has improved specificity for ES, particularly in extraosseous settings [7,12]. However, the definite diagnosis requires confirmation through molecular studies.

Molecular insights

The hallmark of ES is an EWSR1 gene rearrangement. The most common fusion is EWSR1::FLI1, observed in approximately 85% of cases. Less common partners include ERG, ETV1, FEV, and others [1]. Molecular confirmation via FISH or reverse transcription polymerase chain reaction (RT-PCR) is essential for accurate diagnosis and for distinguishing ES from histologic mimics. In the present case, break-apart FISH confirmed an EWSR1 gene rearrangement, consistent with findings in other published cases [1,11].

Treatment and prognosis

The current standard of care involves multimodal therapy, including chemotherapy (VDC/IE), surgical resection, and sometimes radiotherapy. The EURO EWING 2012 (EE2012) phase III trial confirmed the benefit of interval-compressed chemotherapy for newly diagnosed ES [13]. Despite the chemosensitivity of ES, extraosseous cases, particularly those with metastasis at presentation, have significantly worse outcomes.

Reported five-year survival for extraskeletal ES is approximately 38%, compared to 75% for skeletal ES [10]. In adults over 40, Panda et al. [6] reported that the prognosis is further compromised, with a five-year survival of 44.6% [6]. Factors contributing to poor prognosis include larger tumor size, axial location, metastasis at diagnosis, and limited access to intensive therapy. Our patient demonstrated rapid disease progression despite appropriate chemotherapy, emphasizing the aggressive nature of vulvar ES.

## Conclusions

Primary vulvar ES is an extremely rare vulvar tumor. Though it usually presents in adolescents and patients of reproductive age, it can also present in postmenopausal patients. Extraosseous ES should be considered as one of the differential diagnoses for small blue round cell tumors at uncommon sites like the vulva, and IHC is very helpful in arriving at a diagnosis. It is ideal to perform a molecular test for EWSRI 22q12 gene arrangement on such cases for a precise diagnosis. A timely and exact diagnosis has significant therapeutic implications, and early management with multimodal therapy offers benefit.
